# The Associations Between Digital Exclusion and Physical or Cognitive Function in Middle-Aged and Older Adults: Systematic Review and Meta-Analysis

**DOI:** 10.2196/75920

**Published:** 2026-04-23

**Authors:** Jia Yu, Jundan Huang, Shuhan Zhou, Xiao Wan, Qi Xie, Yinan Zhao, Juan Yang, Hui Feng

**Affiliations:** 1 Xiangya School of Nursing, Central South University, No. 172, Tongzipo Road, Yuelu District, Changsha, Hunan, China, 86 15173121969; 2 Hunan Traditional Chinese Medical College, Zhuzhou, Hunan, China; 3 Oceanwide Health Management Institute, Central South University, Changsha, Hunan, China; 4 Hunan Engineering Research Center for Intelligent Medical Care, Central South University, Changsha, Hunan, China

**Keywords:** digital exclusion, internet nonuse, physical function, cognitive function, meta-analysis

## Abstract

**Background:**

Digital exclusion posed a significant challenge, especially in middle-aged and older adults, which affected their health outcomes. However, the evidence regarding the associations of digital exclusion on physical or cognitive function outcomes was controversial, and no systematic review had been performed to synthesize the pooled associations.

**Objective:**

This study aimed to explore the relationship between digital exclusion and physical or cognitive function in middle-aged and older adults.

**Methods:**

We conducted a systematic review and meta-analysis of cohort and cross-sectional studies, including Chinese or English publications retrieved from PubMed, Embase, Web of Science, PsycINFO, Scopus, CNKI, and Wanfang databases up to August 31, 2024. The risk of bias was evaluated using the Newcastle-Ottawa Scale (NOS). The pooled effect size was calculated based on odds ratios (ORs), hazard ratios, risk ratios, and 95% CIs. This study was registered on PROSPERO (CRD42024585459).

**Results:**

Nineteen studies met the inclusion criteria, including 13 cohort studies and 6 cross-sectional studies, which had moderate-to-low risk of bias. The pooled analysis indicated that digital exclusion had prospective associations with decreased basic activities of daily living (incidence rate ratio 1.35, 95% CI 1.12‐1.64, *I*^2^=94.7%) and instrumental activities of daily living (incidence rate ratio 1.46, 95% CI 1.13‐1.89, *I*^2^=96.2%), or cross-sectional association with activities of daily living (OR 1.23, 95% CI 0.41‐3.73, *I*^2^=91%), with no statistical significance in the prospective association with frailty (OR 1.21, 95% CI 0.92‐1.59, *I*^2^=95.2%). There were prospective associations between digital exclusion and dementia (hazard ratio 1.78, 95% CI 1.43‐2.22, *I*^2^=0%), decreased Mini-Mental State Examination scores (OR 1.96, 95% CI 1.39‐2.75, *I*^2^=0%), as well as cross-sectional associations with Mini-Mental State Examination scores (OR 2.90, 95% CI 2.07‐4.07, *I*^2^=0%), and no statistical significance in the prospective association with cognitive impairment (risk ratio 2.08, 95% CI 0.98‐4.44, *I*^2^=78.2%).

**Conclusions:**

Our findings indicated the negative associations of digital exclusion with physical and cognitive functions. Future research and clinical practice should consider designing digital interventions and services that match the physical and cognitive capacities and preferences of middle-aged and older adults, thereby improving digital engagement and reducing the harms associated with digital exclusion. Policies should focus on expanding access, reducing financial barriers, and improving digital literacy. However, due to the presence of heterogeneity and publication bias, the results should be interpreted cautiously.

## Introduction

Population aging has become a global concern, posing significant challenges to public health systems and social development [1]. Although aging is an inevitable biological process, a growing body of research has focused on promoting healthy aging, which emphasizes maintaining and improving intrinsic capacity to enhance overall well-being among middle-aged and older adults [2-4]. The World Health Organization defines healthy aging as “the process of developing and maintaining the functional ability that enables well-being in older age.” [5] This functional ability is underpinned by 2 core components: physical and cognitive functions [6]. Physical function provides the strength, balance, and mobility necessary for action, while cognitive function supports the attention, memory, and decision-making needed to guide those actions effectively and safely [7-10]. Preserving both is essential for maintaining independence and preventing disability in later life, and accumulating evidence suggests that midlife might represent a valuable window for earlier monitoring or intervention [11,12].

Rapid digitalization has profoundly transformed the way individuals access information, communicate, and engage with health and social services, such as monitoring age-related diseases and promoting remote health management [13-15]. However, numerous middle-aged and older adults with substantial health care needs are excluded from digital technologies. They are often unwilling or unable to use digital technology critically, which exacerbates the vicious cycle of digital exclusion and health inequalities [16,17]. The prevalence of digital exclusion varies by country and is especially obvious in older adults, accounting for 21.69% in Denmark, 22.61% in the United Kingdom, 60.78% in Mexico, and 97.15% in China [18]. Digital exclusion currently lacks a unified definition and assessment tool. While some studies define it as the ability to send and receive online messages with difficulty [19], it is most commonly defined and measured as self-reported nonuse of the internet [18,20,21].

Growing evidence suggests that digital exclusion is related to adverse health outcomes, such as functional dependence [22], cognitive impairment [18], and psychological distress [23], ultimately elevating the risk of mortality and exacerbating socioeconomic burdens [24]. The evidence on the negative impact of digital exclusion on psychological well-being is particularly well-documented and has been proven to be associated with higher levels of depression [25,26], loneliness [21,27], and social isolation [28,29] in large cohort studies and systematic reviews.

However, evidence regarding the associations of digital exclusion on physical or cognitive function outcomes was controversial, and no systematic review had been performed to synthesize the pooled associations. Several studies analyzed data from 5 nationally representative cohorts conducted in both developed and developing countries. These studies reported that nonuse of the internet was associated with increased risks of cognitive impairment, functional dependence, and frailty [17,18,30]. However, Liu et al [31] found no statistically significant association between internet nonuse and functional disability in China, and García-Esquinas et al [32] observed a similar nonsignificant relationship between nonuse of computers and frailty in European populations. This inconsistency may arise from differences in study populations, definitions of digital exclusion, measurement methods, and sample sizes. To date, no systematic review has comprehensively synthesized the available evidence or assessed the overall magnitude and direction of these associations. A rigorous synthesis is therefore needed to clarify the impact of digital exclusion on functional health, identify potential sources of heterogeneity, and provide an evidence-based foundation for interventions aimed at promoting healthy aging in digitally marginalized populations.

Therefore, this study aims to systematically identify studies regarding the relationship between digital exclusion and physical or cognitive function in middle-aged and older adults, summarize their findings, and assess the certainty of their evidence to provide a comprehensive understanding of the associations.

## Methods

### Study Design

This review adhered to the PRISMA (Preferred Reporting Items for Systematic Reviews and Meta-Analyses) 2020 statement [33] and was registered on PROSPERO (CRD42024585459; Checklist 1).

### Search Strategies

We searched PubMed, Embase, Web of Science, PsycINFO, Scopus, CNKI, and Wanfang databases, from the inception of the databases to August 31, 2024. Only eligible full texts in English or Chinese were considered for review. Two thematic blocks of keywords were used: “Aged” and “Digital exclusion.” The search terms were (aged or “middle aged” or “older adult*” or aging or “elder*”) AND (“digital exclusion” or “digital inclusion” or “digital divide” or “Internet access” or “Internet use*” or “Internet non-use” or “web usage” or “web use” or “Internet usage” or “digital engagement”). In terms of developing search strategies, we first identified standardized terminology by consulting the Medical Subject Headings (MeSH) database in PubMed. This step ensured the inclusion of entry terms. Subsequently, we examined key published literature to incorporate nonstandardized terms that are conceptually related to digital exclusion, such as “digital divide,” “digital inclusion,” and “digital engagement” [27,34,35]. The detailed search strategies are shown in Multimedia Appendix 1.

### Eligibility Criteria

The inclusion criteria for studies were as follows: (1) middle-aged and older adults as research participants, explicitly aged 45 years and older [36]; (2) digital exclusion was clearly specified as the exposure variable, without redefinition based on dose or frequency; and (3) reporting at least one measure of physical or cognitive function. Specifically, physical function was measured using standardized assessments of functional independence and mobility (eg, activities of daily living [ADLs] and Fried frailty phenotype). Cognitive function was assessed using standardized screening tools (eg, Mini-Mental State Examination [MMSE]) or based on a clinical diagnosis of cognitive impairment or dementia; (4) provision of the odds ratios (ORs), hazard ratios (HRs), risk ratios (RRs), and their corresponding 95% CIs for the associations between digital exclusion and health outcomes; and (5) observational studies.

This study excluded (1) editorials, letters, comments, meeting abstracts, and newspaper articles; (2) studies unable to provide the full text; (3) studies that only reported correlation metrics, such as Pearson *r* or Spearman ρ, without providing data that could be used to calculate ORs, RRs, HRs, and their 95% CIs; and (4) studies that were not published in English or Chinese.

### Study Selection and Data Extraction

Study selection was conducted independently in 2 stages by 2 researchers. A third researcher was invited to resolve the disagreement. Initially, the titles and abstracts of the studies were evaluated in terms of eligibility criteria. In the subsequent stage, full texts of preselected studies were reviewed to confirm their eligibility.

The selected studies were extracted by 2 researchers using a form to extract data on the setting, study design, sample size, basic participant information, digital exclusion assessment tools, categories of digital exclusion, outcome assessment tools, categories of assessment, covariates, and effect sizes of outcomes. Discrepancies between the researchers regarding data extraction were resolved through discussion and consensus with the assistance of a third researcher.

### Methodological Quality Assessment

Two researchers independently assessed the methodological quality of each study using the Newcastle-Ottawa Scale (NOS), a quality assessment tool for both cohort and cross-sectional studies [37]. The tool comprises 8 items with a score range of 0‐9 for cohort studies, and 7 items from 0 to 8 for cross-sectional studies [38], as detailed in Multimedia Appendix 2 [17,18,30,32,39-47] and Multimedia Appendix 3 [19,24,31,48-50]. Initially, we conducted a pilot evaluation of 4 articles (2 cohort studies and 2 cross-sectional studies) to standardize the assessment criteria. Discrepancies between the researchers were discussed, and a third researcher was invited to resolve them collectively.

### Synthesis and Data Analysis

Descriptive analysis was conducted to summarize the characteristics of the studies. For meta-analysis, the associations between digital exclusion and health outcomes were quantitatively synthesized, and effect sizes were measured using RR, OR, HR, and 95% CIs. Separate meta-analyses were conducted for ORs, RRs, and HRs. Effect sizes adjusted for potential confounders were extracted to ensure comparability across studies, allowing them to be regarded as standardized estimates [51]. When the original studies used “digital exclusion” as the reference group, the reported effect sizes were inverted to represent the impact of digital exclusion versus nonexclusion. This conversion was justified by the mathematical principle that inverting a ratio measure was equivalent to swapping the reference and exposure groups [52]. And all conversions were performed with double-checking for accuracy.

The random-effects model was used if significant heterogeneity (*I*^2^>50% or *P*<.1) was found among the included studies; otherwise, the fixed-effect model was used. Due to the inclusion of fewer than 10 studies in the meta-analysis, publication bias analysis was not conducted. The meta-analysis and forest plots were performed using R software (R Core Team) with the main package of “*metafor*.”

### Sensitivity Analysis

Leave-one-out sensitivity analysis was conducted to evaluate the influence of each study on the pooled association estimate. In this procedure, the meta-analysis was repeated multiple times, each time omitting one study and recalculating the pooled association using the remaining studies. When between-study heterogeneity (*I²*) exceeded 50%, a random-effects model was applied for the recalculated estimate [53]. Specifically, a study was considered to have a substantial influence if its exclusion caused the new point estimate to fall outside the 95% CI of the original pooled estimate. A study was also deemed influential if the effect size changed noticeably compared to the primary analysis. Noticeable changes were defined as either a reduction of 25‐50 percentage points or a shift from high heterogeneity (*I*^²^>50%) to low or moderate heterogeneity (*I*^²^≤50%) [54-56]. Due to the limited number of available studies, subgroup analysis based on different definitions, frequencies, or ages could not be performed. All results were presented visually using forest plots, along with corresponding CIs. A statistical significance threshold of *P*<.10 was adopted.

## Results

### Characteristics of the Studies

**Figure 1.**
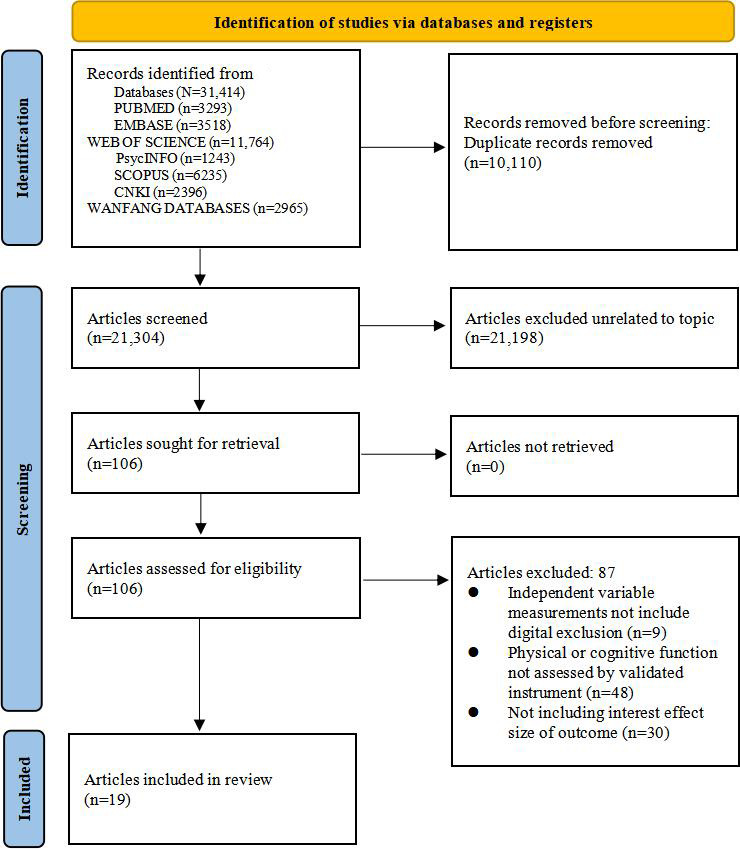
The PRISMA (Preferred Reporting Items for Systematic Reviews and Meta-Analyses) flow diagram.

A total of 31,414 articles were searched from the databases. After excluding duplicate publications (*n*=10,110) and ineligible articles (*n*=21,198), 19 studies were included in this review (Multimedia Appendix 4) [17-19,24,30-32,39-50]. The selection process of the studies was summarized in the PRISMA flow diagram (Figure 1).

Tables1  2 provide the characteristics of the included cohort and cross-sectional studies, respectively. Three studies focused on middle-aged and older adults aged 45 years or older [24,42,45], while the remaining articles specifically concentrated on older adults aged 60 years or older [17-19,30-32,39-41,43,44,46-50]. Thirteen cohort studies [17,18,30,32,39-47] and 6 cross-sectional studies [19,24,31,48-50] were from different regions, including Spain, England, Japan, the United States, China, Europe, Mexico, Brazil, Sweden, and the Netherlands. These studies were conducted between 2012 and 2024, with the sample sizes ranging from 409 to 155,695. The follow-up duration of the cohort studies ranged from 3 to 17.1 years.

**Table 1.** Characteristics of the included cohort studies.

Study	Year	Country	Follow-up (year)	Sample size	Age (years), mean (SD)	Sex (male), n (%)	Digital exclusion definition	Measurements on outcome (outcome)	OR^a^/RR^b^/HR^c^ (95%CI)
Physical function									
García-Esquinas et al [32]	2017	Spain	4	1882	≥60	912 (48.5)	First tertile of the usual number of hours or days spent on computers	Fried frailty phenotype (physical frailty)	OR 0.909 (0.813-1)
García-Esquinas et al [32]	2017	England	4	3989	≥60	1893 (47.5)	First tertile of the usual number of hours or days spent on computers	Fried frailty phenotype (physical frailty)	OR 1.000 (0.952-1.041)
García-Esquinas et al [32]	2017	Spain and England	4	5871	≥60	2805 (47.8)	First tertile of the usual number of hours or days spent on computers	Fried frailty phenotype (physical frailty)	Random-effects meta-analysis OR 1.05 (0.95-1.13)
Li [17]	2024	United States	6	62,932	66.24 (10.28)	25,928 (41.2)	Nonuse of the internet	Frailty index (frailty)	OR 1.299 (1.250-1.351)
Li [17]	2024	China	7	36,866	62.32 (8.37)	17,418 (47.25)	Nonuse of the internet in the past month	Frailty index (frailty)	OR 1.613 (1.429-1.852)
Li [17]	2024	Europe	6	124,926	68.00 (9.75)	54,307 (43.47)	Use the internet less than once a week in the last 7 days	Frailty index (frailty)	OR 2.128 (1.316-3.448)
Li [17]	2024	England	6	27,146	67.65 (9.25)	12,325 (45.40)	Nonuse of the internet	Frailty index (frailty)	OR 1.408 (1.299-1.515)
Li [17]	2024	Mexico	6	34,273	65.15 (9.42)	14,404 (42.03)	Nonuse of the internet	Frailty index (frailty)	OR 1.235 (1.176-1.316)
Li [17]	2024	32 countries	6‐7	155,695	62‐68	66,949 (43)	Nonuse of the internet	Frailty index (frailty)	Random-effects meta-analysis OR 1.389 (1.493-1.266)
Tomioka et al [39]	2024	Japan	3	7913	≥65	3671 (46.4)	Nonuse of the internet	Public long-term care insurance (incident disability)	Several times a year RR 1.205 (0.893-1.639) Several times a month RR 1.031 (0.769-1.389) Several times a week RR 1.205 (0.935-1.538) Almost every day RR 1.493 (1.176-1.887)
Lu et al [30]	2022	United States	6	49,583	72	20,469 (41.3)	Nonuse of the internet	BADLs^d^ and IADLs^e^ (functional ability)	BADLs IRR^f^ 1.31 (1.24-1.37) IADLs IRR 1.54 (1.45-1.64)
Lu et al [30]	2022	England	8	27,338	69	12,853 (47)	Nonuse of the internet	BADLs and IADLs (functional ability)	BADLs IRR 1.21 (1.13-1.30) IADLs IRR 1.25 (1.17-1.33)
Lu et al [30]	2022	Europe	4	96,184	70	42,160 (43.8)	Use the internet less than once a week in the last 7 days	BADLs and IADLs (functional ability)	BADLs IRR 1.43 (1.36-1.51) IADLs IRR 1.43 (1.37-1.49)
Lu et al [30]	2022	China	8	23,342	67	11,261 (48.2)	Nonuse of the internet in the past month	BADLs and IADLs (functional ability)	BADLs IRR 2.04 (1.63-2.55) IADLs IRR 2.45 (1.95-3.09)
Lu et al [30]	2022	Mexico	6	26,968	69	11,854 (44)	Nonuse of the internet	BADLs and IADLs (functional ability)	BADLs IRR 1.07 (1.01-1.13) IADLs IRR 1.07 (1.00-1.14)
Cognitive function									
Krug et al [40]	2019	Brazil	4	1197	≥60	NR^g^	Continued not using the internet	MMSE^h^ (overall cognitive function)	Cognitive loss OR 2.564 (1.136-5.882)
Krug et al [40]	2019	Brazil	4	1197	≥60	NR	Continued not using the internet	MMSE (overall cognitive function)	Cognitive gain OR 0.301 (0.102-0.885)
Wang et al [18]	2024	China	8	7935	67.35 (5.97)	8872 (53.13)	Nonuse of the internet in the past month	Orientation, memory, and executive function tests	OR 2.81 (1.84-4.28)
Wang et al [18]	2024	England	4	6824	70.91 (7.48)	7267 (47.59)	Nonuse of the internet	Orientation, memory, and executive function tests	OR 1.92 (1.70-2.18)
Wang et al [18]	2024	United States	6	13,624	75.30 (7.36)	15,857 (41.04)	Nonuse of the internet	Orientation, memory, and executive function tests	OR 2.48 (2.28-2.71)
Wang et al [18]	2024	Mexican	3	10,470	71.06 (7.59)	7546 (43.41)	Nonuse of the internet	Orientation, memory, and executive function tests	OR 1.92 (1.74-2.12)
Wang et al [18]	2024	Europe	4	23,560	70.52 (7.56)	10,757 (45.53)	Use the internet less than once a week in the last 7 days	Orientation, memory, and executive function tests	OR 2.60 (2.34-2.88)
Berner et al [41]	2019	Sweden	6	2872	66‐96	1190 (41.4)	Nonuse of the internet	MMSE (overall cognitive function)	OR 1.923 (1.25-2.941)
Berner et al [41]	2019	Netherlands	6	683	66‐94	282 (41.3)	Nonuse of the internet	MMSE (overall cognitive function)	OR 1.639 (0.763-3.571)
Cho et al [42]	2023	United States	17.1 (median 7.9)	18,154	55.17 [53.17‐57.25]	7758 (47.36)	Nonuse of the internet	Modified telephone interview for cognitive status (Incident dementia)	HR 1.852 (1.389-2.439)
Williams et al [43]	2020	England	10	3937	61.7(7.9)	1709 (43.4)	Nonuse of the internet	Modified telephone interview for cognitive status (Dementia or cognitive impairment)	Wave 2 RR 1.351 (1.124-1.639) Wave 3 RR 1.282 (1.064-1.538) Wave 4 RR 1.389 (1.163-1.667) Wave 5 RR 1.563 (1.299-1.852) Wave 6 RR 1.515 (1.266-1.786)
Quialheiro et al [47]	2021	Brazil	10	594	≥60	482 (38.6)	Nonuse of the internet	MMSE (overall cognitive function)	RR 3.333 (1.639-6.667)
Almeida et al [44]	2012	Australia	6	5506	75.5 (4.2)	5506 (100)	Nonuse of computers	Western Australian Data Linkage System (dementia)	HR 1.613 (1.235-2.128)
d’Orsi et al [45]	2017	England	10	8238	≥50	3713 (45.07)	Nonuse of the internet	short-form IQCODE^i^ questionnaire (dementia)	HR 1.667 (1.176-2.381)
Nakagomi et al [46]	2021	Japan	3	4232	≥65	1575 (37.2)	Nonuse of the internet or email	Public long-term care insurance (dementia)	Use a few times a month OR 1.190 (0.752-1.887) Use a few times a week OR 1.450 (0.826-2.564) Use almost every day OR 1.176 (0.758-1.852)

a OR: odds ratio.

b RR: risk ratio.

c HR: hazard ratio.

d BADL: basic activity of daily living.

e IADL: instrumental activity of daily living.

f IRR: incidence rate ratio.

g NR: not reported.

h MMSE: Mini-Mental State Examination.

i IQCODE: Informant Questionnaire on Cognitive Decline in the Elderly.

**Table 2.** Characteristics of the included cross-sectional studies.

Study	Year	Country	Sample size	Age (years), mean (SD)	Sex (male), n (%)	Digital exclusion definition	Measurements on outcome	OR^a^/RR^b^/HR^c^ (95% CI)
Physical function								
Wen et al [24]	2023	China	13,474	61.50 (9.30)	6526 (48.43)	Nonuse of the internet	ADLs^d^ (functional ability)	OR 2.083 (1.667-2.564)
Medeiros et al [19]	2012	Brazil	1656	≥60	598 (36.1)	Unable to send or receive online messages	ADLs (functional ability)	RR 1.639 (1.064-2.50)
García-Vigara et al [50]	2022	Spain	409	67.45 (7.81)	0	Nonuse of information and communication technology	Fried frailty phenotype (physical frailty)	OR 10.62 (5.34-21.10)
Cognitive function								
Liu et al [49]	2023	China	10,325	60.32 (9.06)	5698 (55.19)	Nonuse of mobile payment	MMSE^e^ (overall cognitive function)	OR 3.48 (2.27-5.33)
Li et al [48]	2022	China	3020	≥60	1378 (45.6)	Nonuse of the internet	MMSE (overall cognitive function)	MCI OR 2.092 (1.153-3.788) Dementia OR 2.545 (0.601-10.753)
Physical function and cognitive function								
Liu et al [31]	2023	China	5868	68.07 (6.1)	3083 (52.54)	Nonuse of the internet in the last month	BADLs^f^ and Brief Community Screening Instrument for Dementia (functional disability and dementia)	Physical function OR 0.671 (0.357-1.260) Cognitive function OR 19.231 (3.846-90.909)

a OR: odds ratio.

b RR: risk ratio.

c HR: hazard ratio.

d ADL: activity of daily living.

e MMSE: Mini-Mental State Examination.

f BADL: basic activity of daily living.

The assessment tools for digital exclusion showed diversity among the included studies. None of the studies used a validated scale; instead, they all used single-item measures to assess digital exclusion. Most studies (n=14) [17,18,24,30,31,39-43,45-48] defined digital exclusion as nonuse of the internet, while other studies defined digital exclusion as nonuse of computers (n=2) [32,44], nonuse of information and communication technology (n=1) [50], nonuse of mobile payment (n=1) [49], and inability to send or receive online messages (n=1) [19].

Seven studies focused exclusively on physical function [17,19,24,30,32,39,50], while 11 examined cognitive function [18,40-49]. Only 1 study assessed both physical and cognitive outcomes [31]. Physical function was assessed by ADLs (n=3) [19,24,30], Frailty index (n=1) [17], and Fried frailty phenotype (n=2) [32,50], with 1 study obtaining physical function outcomes by linking to data from local government systems [39]. The cognitive function was assessed using numerous cognitive tests, including MMSE (n=5) [40,41,47-49], orientation, memory, and executive function tests (n=1) [18], Modified Telephone Interview for Cognitive Status (n=2) [42,43], short-form Informant Questionnaire on Cognitive Decline in the Elderly (IQCODE) questionnaire (n=1) [45], and Brief Community Screening Instrument for Dementia (n=1) [31], with some studies obtaining cognitive function outcomes by linking to data from local government systems.

### Risk of Bias Assessment

The overall risk of bias assessment of cohort and cross-sectional studies is shown in MultimediaAppendices 2  3.

The overall risk of bias score of cohort studies had a low risk of bias, varying from 7 to 9 points. Regarding the selection bias, all studies included the selection of a nonexposed cohort and the ascertainment of exposure. Similarly, in the comparability and outcome domains, all studies ensured the comparability of cohorts based on design or analysis, assessment of outcomes, and an adequate follow-up period for outcomes to occur. However, 6 studies failed to demonstrate that the outcome of interest was not present at the start of the study [17,18,30,41,43,46], and 4 studies had inadequate cohort follow-up [18,40,41,47].

The overall risk of bias score of cross-sectional studies ranged from 3 to 7 points. All studies clearly stated the study population, had a sample size of more than 300, and assessed the outcome. However, none of the studies reported whether the studied sample had a participation rate of at least 70% (7/10) of the invited individuals.

### Association Between Digital Exclusion and Physical Function

**Figure 2.**
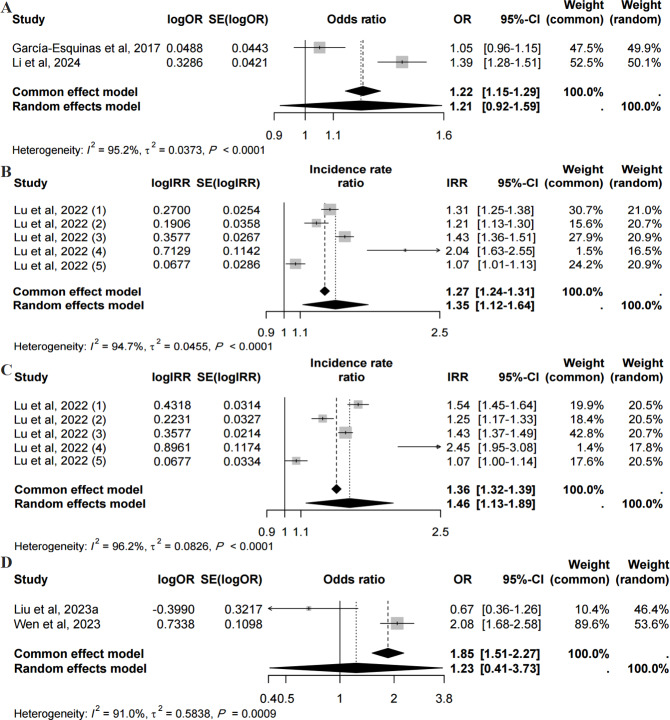
Forest plots of prospective and cross-sectional associations between digital exclusion and physical function [17,24,30-32]. (A) The odds ratio value of the prospective association between digital exclusion and frailty. (B) The incidence rate ratio value of the prospective association between digital exclusion and basic activities of daily living. (C) The incidence rate ratio value of the prospective association between digital exclusion and instrumental activities of daily living. (D) The odds ratio value of the cross-sectional association between digital exclusion and activities of daily living. IRR: incidence rate ratio; OR: odds ratio; RR: risk ratio.

Four studies investigated the prospective association between digital exclusion and frailty, basic activities of daily living (BADLs), instrumental activities of daily living (IADLs), and incident disability [17,30,32,39]. García-Esquinas et al [32] and Li et al [17] investigated the OR value of the association between digital exclusion and frailty. The meta-analysis indicated that there was no statistically significant prospective association between them (OR 1.21, 95% CI 0.92‐1.59, *I*^2^=95.2%; Figure 2A). Lu et al [30] investigated the incidence rate ratio (IRR) value of the association between digital exclusion and BADLs or IADLs. The pooled associations indicated that digital exclusion was associated with decreased BADLs (IRR=1.35, 95% CI 1.12‐1.64, *I*^2^=94.7%; Figure 2B) and IADLs (IRR=1.46, 95% CI 1.13‐1.89, *I*^2^=96.2%; Figure 2C).

Regarding the cross-sectional association, Liu et al [31] and Wen et al [24] analyzed the association between digital exclusion and ADLs. Meta-analysis indicated that there was no significant pooled association between digital exclusion and ADLs (OR 1.23, 95% CI 0.41‐3.73, *I*^2^=91%; Figure 2D). Tomioka et al [39] found an association between internet use and a lower risk of incident disability. While García-Vigara et al [50] revealed that digital exclusion was associated with a higher risk of frailty.

The pooled analysis indicated that digital exclusion had prospective associations with decreased BADLs and IADLs, no statistical significance in the prospective association with frailty, or cross-sectional association with ADLs.

### Association Between Digital Exclusion and Cognitive Function

**Figure 3.**
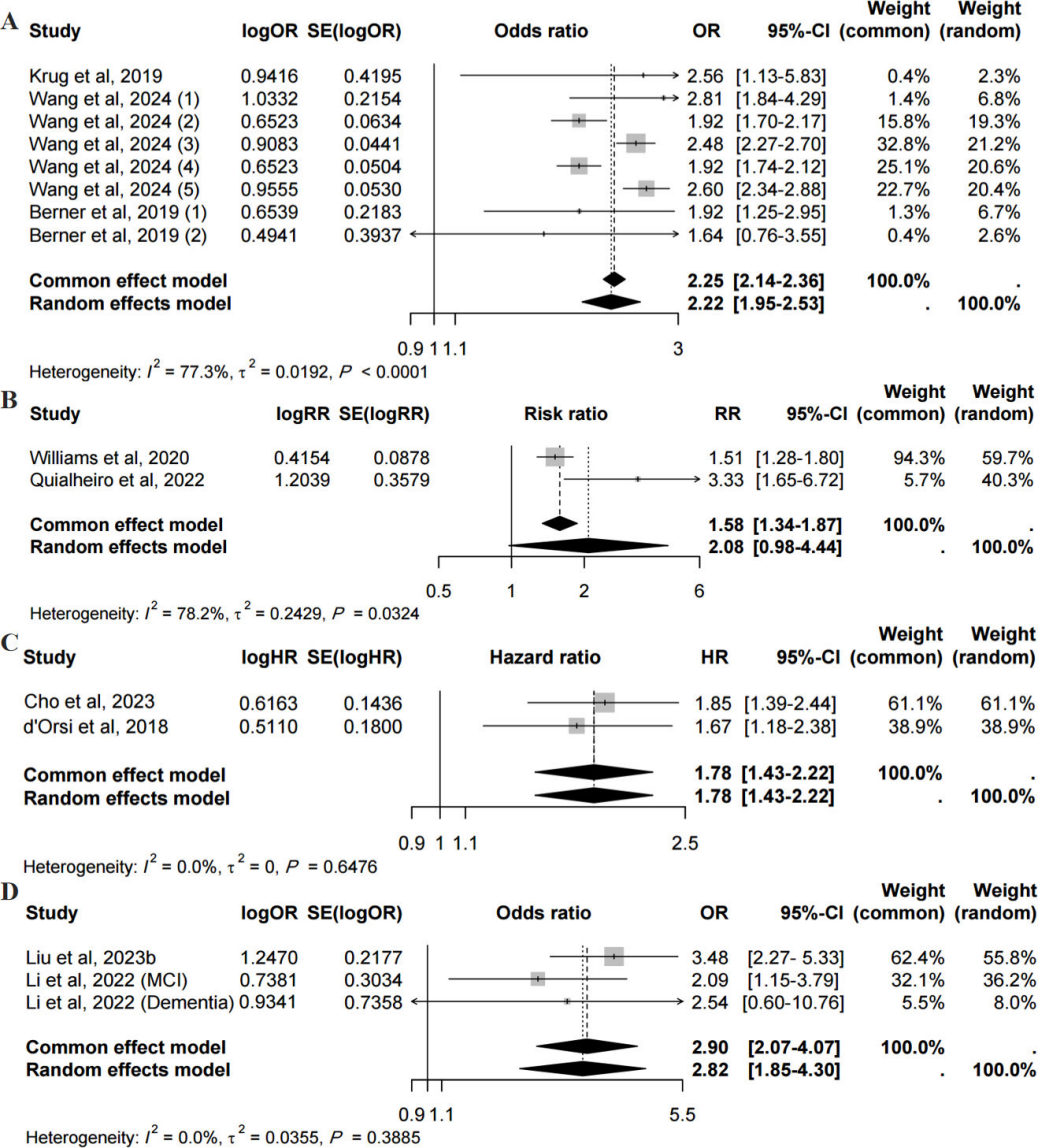
Forest plots of cohort and cross-sectional studies on digital exclusion and cognitive function [18,40-43,45,47-49]. (A) The odds ratio value of the prospective association between digital exclusion and cognitive function. (B) The risk ratio value of the prospective association between digital exclusion and cognitive impairment. (C) The hazard ratio value of the prospective association between digital exclusion and dementia. (D) The odds ratio value of the cross-sectional association between digital exclusion and Mini-Mental State Examination scores. HR: hazard ratio; OR: odds ratio; RR: risk ratio.

Concerning the cohort studies, Krug et al [40], Berner et al [41], and Wang et al [18] explored the OR value of the association between digital exclusion and decreased cognitive function. Meta-analysis of these cohort studies demonstrated that digital exclusion was significantly associated with higher odds of decreased cognitive function (OR 2.22, 95% CI 1.95‐2.53, *I*^2^=77.3%; Figure 3A). Williams et al [43] and Quialheiro et al [47] investigated the relationship between digital exclusion and cognitive impairment. However, meta-analysis of 2 studies [43,47] found no statistically significant association (RR 2.08, 95% CI 0.98‐4.44, *I*^2^=78.2%; Figure 3B). Cho et al [42] and d’Orsi et al [45] analyzed the HR value of the association between digital exclusion and dementia. Meta-analysis demonstrated a significant association between them (HR 1.78, 95% CI 1.43‐2.22, *I*^2^=0%; Figure 3C).

In terms of the cross-sectional studies, a meta-analysis of 2 studies [48,49] showed that digital exclusion was associated with a decline in MMSE scores (OR 2.90, 95% CI 2.07‐4.07, *I*^2^=0%; Figure 3D).

There were prospective associations between digital exclusion and dementia and decreased cognitive function, as well as cross-sectional associations with MMSE scores, but no statistically significant prospective association with cognitive impairment.

### Sensitivity Analysis

**Figure 4.**
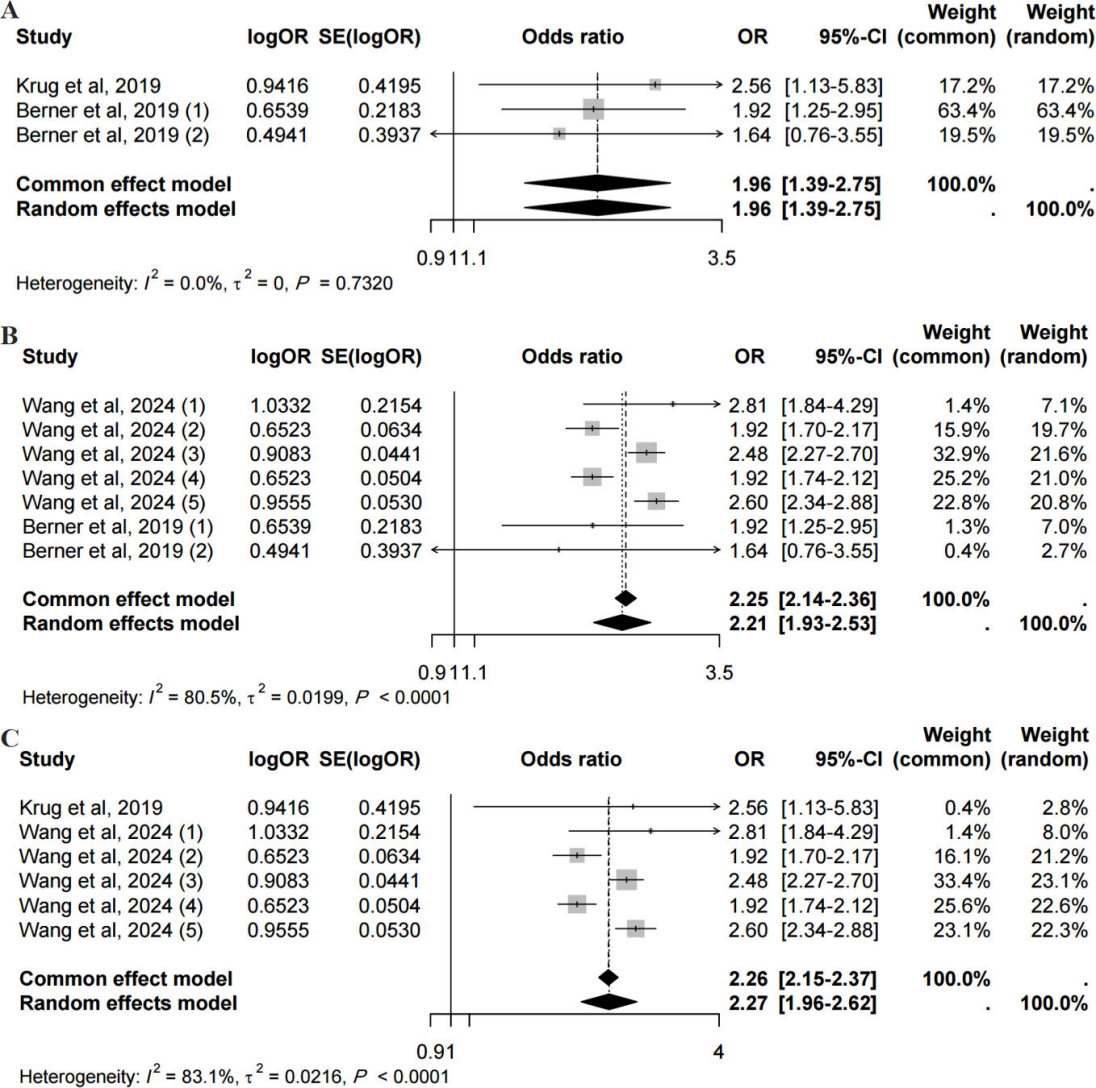
Forest plots of cohort studies on digital exclusion and cognitive functioning [18,40,41]. (A) The odds ratio value of the prospective association between digital exclusion and cognitive function, including studies of Krug et al [40] and Berner et al [41]. (B) The odds ratio value of the prospective association between digital exclusion and cognitive function, including studies of Wang et al [18] and Berner et al [41]. (C) The odds ratio value of the prospective association between digital exclusion and cognitive function, including studies of Krug et al [40] and Wang et al [18]. OR: odds ratio.

Due to the limited number of studies, sensitivity analysis for the association between digital exclusion and physical function was not performed. In the sensitivity analysis of the prospective association between digital exclusion and cognitive function, a leave-one-out approach was applied, and only minor changes were observed in the results, indicating the robustness of our findings (Figure 4).

The sensitivity analysis revealed only minor variations in the results, confirming the stability of our findings, as evidenced by the prospective association between digital exclusion and cognitive function.

## Discussion

### Summary of Evidence

To the best of our knowledge, this study presents the first systematic review and meta-analysis to comprehensively evaluate the associations of digital exclusion on both physical and cognitive functions across multiple countries. Synthesizing evidence from 13 cohorts [17,18,30,32,39-47] and 6 cross-sectional studies [19,24,31,48-50], we found that digital exclusion was prospectively associated with declines in IADLs and BADLs, as well as increased risks of incident disability, cognitive impairment, and dementia, and was cross-sectionally related to lower MMSE scores. However, no statistically significant prospective association was observed between digital exclusion and frailty.

### Digital Exclusion and Physical Function

Consistent with previous studies [30,39], the meta-analysis identified a negative prospective association between digital exclusion and physical function. Several underlying explanations may account for the associations observed in this study. First, digital exclusion was associated with a minimal chance of using eHealth [57] and limited access to acquire digital preventive and therapeutic health services [58,59]. Second, it might cause a decrease in physical exercise behavior among middle-aged and older adults [60], which can lead to a reduction in skeletal muscle mass, strength, and physical mobility [61], ultimately accelerating the deterioration of physical functions and creating a vicious cycle [62-64]. Additionally, middle-aged and older adults experiencing digital exclusion were prone to dopamine imbalance and induced negative emotions such as loneliness and social isolation [65,66], increasing the risk of incident disability [67].

The associations of digital exclusion on frailty remained debated, and the meta-analysis found no statistically significant prospective association. Frailty is influenced by numerous factors such as age, gender, physiological changes, and other factors [68-70]. Furthermore, the influence of digital exclusion on frailty is likely indirect. It may operate through social isolation, limited access to health information, and reduced physical or cognitive engagement, which accumulate gradually over time [32]. These factors could mask a true association in short-term analyses. In addition, the limited number of studies included in the meta-analysis provided only a modest evidence base. The association between digital exclusion and frailty requires further validation through standardized longitudinal studies with longer follow-up periods and consistent assessments of both digital exclusion and frailty.

Meanwhile, digital exclusion did not demonstrate a cross-sectional association with physical function. One explanation for this inconsistency was the influence of confounding factors. It was also possible that socioeconomic and health-related factors masked the cross-sectional relationship between digital exclusion and physical function. Wen et al [24] reported a cross-sectional association between digital exclusion and physical function without adjusting for confounders [71], whereas Liu et al [31] found no such association after adjustment. In addition, the cross-sectional design was not well-suited to detect the slower impact of digital exclusion on physical function, which emerged gradually through changes in health behaviors and disease management. These findings underscore the need for longitudinal designs to clarify temporal relationships and potential causal pathways.

### Digital Exclusion and Cognitive Function

Findings of this study regarding the relationship between digital exclusion and cognitive function broadly aligned with previous research [72-75]. Apart from the aforementioned impact of health care resources and negative psychological emotions, digital exclusion may influence cognitive function through several mechanisms. It not only reduces engagement in cognitively stimulating activities that build cognitive reserve but may also promote negative emotions, which can contribute to neurovascular dysfunction [73,76-78]. Additionally, it is linked to structural brain changes such as reduced volume of the globus pallidus [48].

However, it was noteworthy that no statistical prospective association was observed between digital exclusion and cognitive impairment. This discrepancy could be attributed to several methodological variations across studies. For instance, the studies by Williams et al [43] and Quialheiro et al [47] both investigated populations in the United Kingdom with similar follow-up durations. But the latter had a much smaller sample size (n=594 vs 3937), which could have limited its statistical power and affected the ability to detect significant associations. Furthermore, the 2 studies [43,47] used different assessment tools, which might contribute to the considerable heterogeneity in the meta-analysis.

### Dose-Response Relationship and Types of Digital Exclusion

Digital exclusion or excessive internet use both had negative impacts on health outcomes [79-81]. These findings revealed “two sides of the same coin” and emphasized the importance of the frequency of internet use. In terms of the dose-response relationships, previous studies have found an inverted U-shaped association between internet use and cognitive function [42]. In the large-scale prospective cohort study of Cho et al [42], using the internet almost weekly was most beneficial to cognitive function. Additionally, Williams et al [43] and Liao et al [82] have indicated that different types of internet use exert varying impacts on physical and cognitive functions. Previous research showed diverse internet activities benefit cognition more than single ones [83]. However, the optimal types, combinations, and intensity of digital engagement for enhancing physical and cognitive functions in middle-aged and older adults remain unclear. Future research should investigate these factors across different age groups and geographic regions to tailor digital interventions to the needs and capacities of diverse populations.

### Different Definitions and Assessment Tools of Digital Exclusion

The prevalence of digital exclusion varies substantially across studies due to differences in national populations, as well as inconsistencies in the definition and assessment tools of digital exclusion among middle-aged and older adults. Some studies had suggested that digital exclusion constituted a part of social exclusion [30], as digital technology marginalized older adults from the digital world [84-86]. It might be associated with a perceived lack of knowledge, inability to access necessary technology and services, and barriers posed by mental health difficulties [85]. The absence of a unified definition and assessment tool not only reduced the reliability and generalizability of our findings due to high heterogeneity but also hindered the development of effective policies and interventions to address digital exclusion. Future research should focus on establishing a consensus-based conceptual framework and developing rigorously validated assessment instruments of digital exclusion. To ensure global applicability, these tools should be culturally and contextually adaptable, with careful attention to cross-cultural validation that ensures their relevance across diverse populations and settings.

### Potential of Assessments Based on Digital Biomarkers

Current assessments of physical and cognitive function in the included studies rely largely on scales that capture activities of daily living, memory, orientation, and related abilities [18,30]. While these tools provide valuable information on functional status, they are largely subjective and may be influenced by self-report bias, assessor variability, or ceiling and floor effects [30,87]. Moreover, evidence directly linking these functional scales to objective biomarkers remains limited. Gotti et al [88] found that inflammatory and immune biomarkers changed significantly after internet-based psychological interventions, demonstrating that digital engagement can trigger measurable biological changes. This evidence presented a promising avenue for exploring underlying health mechanisms through objective biological markers. Advances in digital health further extended this possibility [89]. Chan et al [90] developed Watch Walk wrist-worn algorithms that accurately quantify gait speed [90]. This precision allowed large-scale use and turned routine movements into scalable digital biomarkers. Fan et al [70] demonstrated that integrating wearable gait data with machine learning models can substantially improve frailty prediction [91]. Together, these developments illustrate an emerging capacity to collect continuous or longitudinal data in real-world settings, enabling earlier detection of functional decline and a more nuanced understanding of how digital behaviors may shape health trajectories over time.

### Mitigating Digital Exclusion for Middle-Aged and Older Adults

Although growing evidence supports the use of digital technologies to improve physical and cognitive function [92,93], reconciling digital exclusion and digital adoption remains a major challenge. To bridge this divide, it is essential to implement strategies that enhance digital access, strengthen digital literacy, and support digital assimilation [94]. Facilitating the introduction and adoption of user-friendly technologies, particularly those tailored to the needs of older adults, can play a critical role in ensuring equitable digital participation. A growing body of research highlighted co-design as a key approach [95-97]. Chan et al [98] used co-design workshops to involve older adults, health care providers, and community staff in refining a mobile health prototype, ensuring better usability and engagement. Digital navigators can offer literacy training, resolve technical issues, and guide app use, thereby improving technology adoption and integration into care [99]. Beyond design, social support also plays a crucial role. Hänninen et al [100] found that younger family members often act as “warm experts,” offering practical, personalized support that helps older adults manage digital tools with greater confidence.

### Limitations and Implications

First, the generalizability of our findings is subject to certain constraints. Our meta-analysis was primarily restricted to studies published in English and Chinese, as these were the languages most accessible to the research team. Under the constraints of time and resources, conducting translations of studies in other languages was not feasible. This limitation may hinder the applicability of the results to regions where other languages are dominant or where cultural and socioeconomic contexts differ significantly. Additionally, the overall number of available studies focusing specifically on digital exclusion remains limited, particularly for middle-aged populations. Most included studies predominantly involved older adults, resulting in inadequate representation of middle-aged cohorts. Due to the limited number of included studies, it is hard to follow different age, sex, education level, income, or rural-urban residence groups to conduct subgroup analyses. This restricts the broader generalization of our conclusions across middle-aged and older adults. Future research should aim to incorporate more diverse geographical and linguistic sources, as well as prioritize well-defined studies involving middle-aged populations, to enhance the generalizability and age-specific understanding of digital exclusion.

Second, our meta-analysis is influenced by the substantial heterogeneity observed in some pooled estimates. This heterogeneity mainly arose from clinical diversity, including variations in population characteristics across different countries and income levels. Methodological diversity also contributed to heterogeneity, including differences in how digital exclusion was assessed across the large cohort studies, restriction to risk-based effect measures, variations in sample sizes, and inconsistent adjustment for covariates. By restricting quantitative synthesis to risk-based effect measures, our findings primarily reflect associations framed within an epidemiological risk perspective and may not fully capture evidence derived from correlation-based analyses using continuous measures. We performed sensitivity analyses to test the robustness of the findings and to identify key sources of heterogeneity. However, the limited number of studies in certain subgroups restricted more detailed investigations. If enough studies are available, conducting a subgroup analysis based on different definitions is informative. In the future, it is necessary to unify the definition of digital exclusion and develop recognized tools for assessment.

Third, the impact of the types and frequency of digital exclusion on physical and cognitive functions could not be explored. Future in-depth research could explore the dose-response relationships of various frequencies and types of digital exclusion on physical and cognitive functions.

Fourth, the exclusion of gray literature might lead to publication bias. It is advisable to search specialized gray literature databases when time, resources, and evidence permit.

### Conclusions

Our findings emphasize the negative associations between digital exclusion and physical or cognitive functions. Although our results do not directly address optimal patterns of digital engagement, it is noted that moderate and diverse digital use may be more beneficial than either nonuse or overuse for maintaining these functions. In clinical practice, integrating digital tools into interventions can support physical and cognitive health when tailored to users’ abilities and needs. For policymakers, efforts should focus on expanding internet access, reducing financial barriers, and improving digital literacy through training and awareness programs. For future studies, the need for cautious interpretation and further validation are needed due to variations across studies, assessment tools, and populations.

## Supplementary material

10.2196/75920 Checklist 1 PRISMA checklist.

10.2196/75920 Multimedia Appendix 1 Search strategy.

10.2196/75920 Multimedia Appendix 2 The overall risk of bias assessment of cohort studies.

10.2196/75920 Multimedia Appendix 3 The overall risk of bias assessment of cross-sectional studies.

10.2196/75920 Multimedia Appendix 4 The detailed information of the included studies.

## Abbreviations

ADL: activity of daily living
BADL: basic activity of daily living
HR: hazard ratio
IADL: instrumental activity of daily living
IQCODE: Informant Questionnaire on Cognitive Decline in the Elderly
IRR: incidence rate ratio
MeSH: Medical Subject Headings
MMSE: Mini-Mental State Examination
NOS: Newcastle-Ottawa Scale
OR: odds ratio
PRISMA: Preferred Reporting Items for Systematic Reviews and Meta-Analyses
RR: risk ratio
WHO: World Health Organization
