# Supplementary material for: The Associations Between Digital Exclusion and Physical or Cognitive Function in Middle-Aged and Older Adults: Systematic Review and Meta-Analysis
Source: JMIR Aging. 2026 Apr 23;9:e75920. doi: 10.2196/75920 (PMC13105444; doi:10.2196/75920)
Supplement: Multimedia Appendix 1 [file aging-v9-e75920-s002.docx]

**Multimedia Appendix 1. Search strategy.**

PubMed

| #1 | ("Aged"[Mesh] OR "Middle Aged"[Mesh]) OR ("older adult*"[Title/Abstract] OR "aging"[Title/Abstract] OR "elder*"[Title/Abstract] OR "aged"[Title/Abstract] OR "middle aged"[Title/Abstract]) | 6,394,273 |
| --- | --- | --- |
| #2 | ("digital exclusion"[Title/Abstract] OR "digital inclusion"[Title/Abstract] OR "digital divide"[Title/Abstract] OR "internet access"[Title/Abstract] OR "Internet use*"[Title/Abstract] OR "Internet non-use"[Title/Abstract] OR "web usage"[Title/Abstract] OR "web use"[Title/Abstract] OR "Internet usage"[Title/Abstract] OR "digital engagement"[Title/Abstract]) OR ("Internet Use"[Mesh]) | 9,964 |
| #3 | #1 AND #2 | 3,293 |

Embase

| #1 | 'aged'/exp | 4,036,692 |
| --- | --- | --- |
| #2 | 'middle aged'/exp | 2,409,442 |
| #3 | aged:ab,ti OR 'middle aged':ab,ti OR 'older adult*':ab,ti OR aging:ab,ti OR elder*:ab,ti | 1,751,150 |
| #4 | #1 OR #2 OR #3 | 6,425,325 |
| #5 | 'internet use'/exp | 1,501 |
| #6 | 'digital exclusion':ab,ti OR 'digital inclusion':ab,ti OR 'digital divide':ab,ti OR 'internet access':ab,ti OR 'internet use*':ab,ti OR 'internet non-use':ab,ti OR 'web usage':ab,ti OR 'web use':ab,ti OR 'internet usage':ab,ti OR 'digital engagement':ab,ti | 11,665 |
| #7 | #5 OR #6 | 12,170 |
| #8 | #4 AND #7 | 3,518 |

Web of science

| #1 | TS=(aged OR "middle aged" OR "older adult*" OR aging OR elder*) | 11,454,571 |
| --- | --- | --- |
| #2 | TS=("digital exclusion" OR "digital inclusion" OR "digital divide" OR "internet access" OR "Internet use*" OR "Internet non-use" OR "web usage" OR "web use" OR "Internet usage" OR "digital engagement") | 45,516 |
| #3 | #1 AND #2 | 11,764 |

PsycINFO

| S1 | abstract(aged OR "middle aged" OR "older adult*" OR aging OR elder*) OR title(aged OR "middle aged" OR "older adult*" OR aging OR elder*) | 442,744 |
| --- | --- | --- |
| S2 | abstract("digital exclusion" OR "digital inclusion" OR "digital divide" OR "internet access" OR "Internet use*" OR "Internet non-use" OR "web usage" OR "web use" OR "Internet usage" OR "digital engagement") OR title("digital exclusion" OR "digital inclusion" OR "digital divide" OR "internet access" OR "Internet use*" OR "Internet non-use" OR "web usage" OR "web use" OR "Internet usage" OR "digital engagement") | 9,081 |
| S3 | [S1] AND [S2] | 1,243 |

Scopus

| #1 | ( TITLE-ABS-KEY ( aged ) OR TITLE-ABS-KEY ( "middle aged" ) OR TITLE-ABS-KEY ( "older adult*" ) OR TITLE-ABS-KEY ( aging ) OR TITLE-ABS-KEY ( elder* ) ) | 7,650,348 |
| --- | --- | --- |
| #2 | ( TITLE-ABS-KEY ( "digital exclusion" ) OR TITLE-ABS-KEY ( "digital inclusion" ) OR TITLE-ABS-KEY ( "digital divide" ) OR TITLE-ABS-KEY ( "internet access" ) OR TITLE-ABS-KEY ( "Internet use*" ) OR TITLE-ABS-KEY ( "Internet non-use" ) OR TITLE-ABS-KEY ( "web usage" ) OR TITLE-ABS-KEY ( "web use" ) OR TITLE-ABS-KEY ( "Internet usage" ) OR TITLE-ABS-KEY ( "digital engagement" ) ) | 56,475 |
| #3 | #1 AND #2 | 6,235 |

CNKI

|  | （主题：数字鸿沟 + 数字排斥 + 数字包容 + 互联网使用）AND（主题：中年 + 老年 + 老人） | 2,396 |
| --- | --- | --- |

Wanfang

|  | （中英文扩展&主题词扩展）： 主题:(数字鸿沟 or 数字排斥 or 数字包容 or 互联网使用) and 主题:(中年 or 老年 or 老人) | 2,965 |
| --- | --- | --- |
