# Supplementary material for: The Associations Between Digital Exclusion and Physical or Cognitive Function in Middle-Aged and Older Adults: Systematic Review and Meta-Analysis
Source: JMIR Aging. 2026 Apr 23;9:e75920. doi: 10.2196/75920 (PMC13105444; doi:10.2196/75920)
Supplement: Multimedia Appendix 2 [file aging-v9-e75920-s003.docx]

**Multimedia Appendix 2. The overall risk of bias assessment of cohort studies.**

| **Cohort studies** | **Risk of bias** | | | | | | | | |
| --- | --- | --- | --- | --- | --- | --- | --- | --- | --- |
|  | **Selection** | | | | **Comparability** | **Outcome** | | | **Total score** |
|  | **Represent-ativeness of the Exposed Cohort** | **Selection of the Non-Exposed Cohort** | **Ascertai-nment of Exposu-re** | **Demonstration That Outcome of Interest Was Not Present at Start of Study** | **Comparability of Cohorts on the Basis of the Design or Analysis** | **Assessment of Outcome** | **Was Follow-Up Long Enough for Outcomes to Occur** | **Adequacy of Follow Up of Cohorts** |  |
| García-Esquinas et al, 2017 [32] | 1 | 1 | 1 | 1 | 2 | 1 | 1 | 1 | 9 |
| Li et al, 2024 [17] | 1 | 1 | 1 | 0 | 2 | 1 | 1 | 1 | 8 |
| Berner et al, 2019 [41] | 1 | 1 | 1 | 0 | 2 | 1 | 1 | 0 | 7 |
| Tomioka et al, 2024 [39] | 0 | 1 | 1 | 1 | 2 | 1 | 1 | 1 | 8 |
| Williams et al, 2020 [43] | 1 | 1 | 1 | 0 | 2 | 1 | 1 | 1 | 8 |
| Lu et al, 2022 [30] | 1 | 1 | 1 | 0 | 2 | 1 | 1 | 1 | 8 |
| Almeida et al, 2012 [44] | 0 | 1 | 1 | 1 | 2 | 1 | 1 | 1 | 8 |
| Krug et al, 2019 [40] | 1 | 1 | 1 | 1 | 2 | 1 | 1 | 0 | 8 |
| Cho et al, 2023 [42] | 1 | 1 | 1 | 1 | 2 | 1 | 1 | 1 | 9 |
| d’Orsi et al, 2017 [45] | 1 | 1 | 1 | 1 | 2 | 1 | 1 | 1 | 9 |
| Nakagomi et al, 2021 [46] | 1 | 1 | 1 | 0 | 2 | 1 | 1 | 1 | 8 |
| Quialheiro et al, 2021 [47] | 1 | 1 | 1 | 1 | 2 | 1 | 1 | 0 | 8 |
| Wang et al, 2024 [18] | 1 | 1 | 1 | 0 | 2 | 1 | 1 | 0 | 7 |
