# Supplementary material for: The Associations Between Digital Exclusion and Physical or Cognitive Function in Middle-Aged and Older Adults: Systematic Review and Meta-Analysis
Source: JMIR Aging. 2026 Apr 23;9:e75920. doi: 10.2196/75920 (PMC13105444; doi:10.2196/75920)
Supplement: Multimedia Appendix 3 [file aging-v9-e75920-s004.docx]

**Multimedia Appendix 3. The overall risk of bias assessment of cross-sectional studies.**

| **Cross-Sectional studies** | **Risk of bias** | | | | | | | |
| --- | --- | --- | --- | --- | --- | --- | --- | --- |
|  | **Selection** | | | | **Comparability** | **Outcome** | | **Total score** |
|  | **Representativeness of the sample** | **Sample size（＞300）** | **Ascertainment of exposure** | **Adequate response**  **rate (70%), refused**  **described** | **Confounding factors are controlled** | **Assessment of outcome** | **Statistical test** |  |
| Liu et al, 2023 [31] | 1 | 1 | 1 | 0 | 2 | 1 | 1 | 7 |
| Li et al, 2022 [48] | 1 | 1 | 1 | 0 | 2 | 1 | 1 | 7 |
| Liu et al, 2023 [49] | 1 | 1 | 1 | 0 | 2 | 1 | 1 | 7 |
| Wen et al, 2023 [24] | 1 | 1 | 0 | 0 | 0 | 1 | 0 | 3 |
| Medeiros et al, 2012 [19] | 1 | 1 | 1 | 0 | 2 | 1 | 1 | 7 |
| [García-Vigara](https://pubmed.ncbi.nlm.nih.gov/?sort=date&term=Garc%C3%ADa-Vigara+A&cauthor_id=34275701) et al, 2022 [50] | 1 | 1 | 1 | 0 | 2 | 1 | 1 | 7 |

Reference: Ho PJ, Gernaat SA, Hartman M, Verkooijen HM. Health-related quality of life in Asian patients with breast cancer: a systematic review. BMJ open. 2018 Apr 1;8(4):e020512.
