# Supplementary material for: The Associations Between Digital Exclusion and Physical or Cognitive Function in Middle-Aged and Older Adults: Systematic Review and Meta-Analysis
Source: JMIR Aging. 2026 Apr 23;9:e75920. doi: 10.2196/75920 (PMC13105444; doi:10.2196/75920)
Supplement: Multimedia Appendix 4 [file aging-v9-e75920-s005.docx]

**Multimedia Appendix 4. The detailed information of the included studies.**

| Study | Setting | Study design, follow-up | Participants, sample size, mean age (SD), sex (male percent) | Measurements on digital exclusion, classification | Outcome/physical or cognitive function | Measurements on outcome, classification | Adjustment | Findings, adjusted effect sizes (β/OR/RR/HR) | Other important information |
| --- | --- | --- | --- | --- | --- | --- | --- | --- | --- |
| García-Esquinas et al, 2017 [32] | Spain and England | Cohort study, 4 years | Seniors-ENRICA cohort: 1882; 60+, 912(48.5%)  ELSA: 3989; 60+, 1893(47.5%) | Participants were asked to recall the usual number of hours/day spent on computer. | Physical function: frailty | Fried’s frailty phenotype, frail or non-frail | age, sex, educational level, body mass index (<25, 25-29.9, ≥30 kg/m2), tobacco (never-, ex-, current-smoker), total energy intake (kcal/day), MEDAS index, physical activity, cancer, diabetes, cardiovascular disease, osteomuscular disease and chronic respiratory disease | Seniors-ENRICA cohort: refer to 0, T2: 0.1-0.4 (M) or 0.1-0.6 (W) OR 0.28, 95%CI: (0.07,1.18), T3: >0.4 (M) or>0.6 (W) OR 0.81, 95%CI: (0.38,1.71)  ELSA: refer to 0, Use (>0.1 h/day) OR 0.64, 95%CI: (0.43,0.95) |  |
| Li et al, 2024 [17] | 32 countries | Multicohort study, 6-7 years | HRS: 23,074, 66.24 (10.28), 25,928 (41.20);  ELSA: 9,869, 67.65 (9.25), 12,325 (45.40);  SHARE: 87,517, 68.00 (9.75), 54,307 (43.47);  CHARLS: 17,690, 62.32 (8.37), 17,418 (47.25);  MHAS: 17,545, 65.15 (9.42), 14,404 (42.03) | The response “yes” (HRS, CHARLS, SHARE, and MHAS) or a frequency of at least once a week (ELSA) was classified as internet use, while the response “no” or a frequency of less than once a week was defined as digital exclusion. | Physical function: frailty | Frailty index, frailty or non-frailty | age, gender, educational levels, work for payment, married or partnered, household wealth, smoking, drinking, and co-residence with children | Refer to none, internet use: OR: 0.72 (0.67—0.79) | Generalized estimating equations models, random effects meta-analysis, COX regression, and mediation analysis |
| [García-Vigara](https://pubmed.ncbi.nlm.nih.gov/?sort=date&term=Garc%C3%ADa-Vigara+A&cauthor_id=34275701) et al, 2022 [56] | Spain | Cross-sectional study | postmenopausal midlife and older women, 409, 67.45 (7.81), 0 | Non-use of information and communication technology (including tablets, computers or smartphones) | Physical function: frailty | Fried’s frailty phenotype, frail, pre-frail, robust | age, height, weight, BMI, waist circumference, comorbidities, marital status, financial situation and education level | Refer to any ICT use, none: OR: 10.62 (5.34, 21.10) |  |
| Tomioka et al, 2024 [45] | Japan | Longitudinal study, 3 years | 7913, 65+, 3671(46.4%) | The question about how often do you use the Internet, “almost every day,”“several times a week,” “several times a month,” “several times a year,” or “none.” | Physical function | Public LTCI with a strong correlation with the Barthel index | age, gender, family structure, perceived economic situation, education, chronic medical conditions, body mass index, dietary variety, working status, walking time, and cognitive functioning | Refer to none, several times a year: cumulative incidence ratio 0.83 (0.61–1.12), Several times a month: 0.97 (0.72–1.30), Several times a week: 0.83 (0.65–1.07), Almost every day: 0.67 (0.53–0.85)* | Poisson regression |
| Lu et al, 2022 [30] | 23 countries | Multicohort study, 8 years | HRS: 49583, 72 (65–78), 20,469 (41.3);  ELSA: 27338, 69 (64–76), 12,853 (47.0);  SHARE: 96184, 70 (12–77), 42,160 (43.8);  CHARLS: 23342, 67 (63–72), 11,261 (48.2);  MHAS: 26968, 69 (65–76), 11,854 (44.0) | The response “no” (HRS, SHARE, CHARLS, and MHAS) or a frequency of less than once a week (SHARE) was categorized as digital exclusion. | Physical function | Basic ADLs and IADLs. The score of more than 0 was defined as difficulties in BADL or IADL and 0 as no difficulties. | demographics (age level and gender), socio-economic positions (education, labour force status, household wealth level), living arrangements (marital status and co-residence with children), lifestyles (smoking, drinking), and the presence of chronic conditions (ever had hypertension, stroke, cancer), as well as mental symptoms (depressive symptoms, cognitive impairment) | Refer to use, digital exclusion HRS: BADL difficulty IRR 1.54 (1.24–1.37), IADL 2.48, (1.45–1.64)  ELSA: BADL 1.21 (1.13–1.30), IADL 1.25 (1.17–1.33)  SHARE: BADL 1.43 (1.36–1.51), IADL 1.43 (1.37–1.49);  CHARLS: BADL 2.04 (1.63–2.55), IADL 2.45 (1.95–3.09)  MHAS: BADL 1.07 (1.01–1.13), IADL 1.07 (1.00–1.14) |  |
| Krug et al, 2019 [46] | Brazil | Cohort study, 4 years | 1705, aged 60 years or older, - | Internet use after four years, assessed in a longitudinal way (continued not using the Internet; stopped using the Internet; started using the Internet; continued using the Internet). | Cognitive function | MMSE, gain/loss of four or more points | gender (female, male); age (complete years); per capita household income in Brazilian reais (R$) at the time of interview; schooling (years of schooling); and cognitive decline at baseline | cognitive improvement: continued using the Internet (OR = 3.32; 95%CI 1.13 - 9.76) compared to those who have never used it.  cognitive decrease: continued using the Internet (OR = 0.39 (0.17 – 0.88)) compared to those who have never used it. |  |
| Wang et al, 2024 [18] | 23 countries | Multicohort study, 3-7 years | CHARLS: 7935, 67.35 (5.97), 8872 (53.13%)  ELSA: 6824, 70.91 (7.48), 7267 (47.59%)  HRS: 13624, 75.30 (7.36), 15857 (41.04%)  MHAS: 10470, 71.06 (7.59), 7546 (43.41%)  SHARE: 23560, 70.52 (7.56), 10757 (45.53%) | The responses “no” (CHARLS, HRS, MHAS and SHARE) or “less than once a week” (SHARE) were classified as digital exclusion. | Cognitive function | three cognitive function tests: orientation, memory, and executive function, and through aging associated cognitive decline (AACD) to defne cognitive impairment, namely at least one standard deviation (SD) below the age norm | Age, gender, education, labour force status, household wealth, married or partnered，co-residence with children，smoking, drinking, hypertension, stroke, cancer, and depressive symptom | Refer to use, digital exclusion  CHARLS: cognitive impairment OR 2.81(1.84, 4.28)  ELSA: cognitive impairment OR 1.92(1.70, 2.18)  HRS: cognitive impairment OR 2.48(2.28, 2.71)  MHAS: cognitive impairment OR 1.92 (1.74, 2.12)  SHARE: cognitive impairment OR 2.60 (2.34, 2.88) |  |
| Berner et al, 2019 [47] | Sweden and the Netherlands | Longitudinal study, 6 years | Sweden: 2872, 66-96, 1190(41.4%);  Netherlands: 683, 66-94, 282(41.3%) | The question about internet use was asked in a way to garner a yes or no response from the subject. | Cognitive function decline | MMSE; decline or not decline | gender, age, education, functional limitations, and living situation | Refer to no use, OR 0.54, 95%CI: (0.37, 0.78) |  |
| Cho et al, 2023 [48] | US | Cohort study, maximum of 17.1 (median = 7.9) years | 18154, 55.17 [53.17–57.25], 7758 (47.36) | “Do you regularly use the World Wide Web, or the Internet, for sending and receiving e-mail or for any other purpose?” Participants could answer Yes or No. | Cognitive function | Modified Telephone Interview for Cognitive Status (TICSm), individuals who scored less than 7 out of a total score of 27 in any given wave as having incident dementia | baseline TICSm score, self-reported health, age, household income, marital status, and the region of residence | Refer to no-regular use, HR 0.54 [0.41-0.72] |  |
| Williams et al, 2020 [49] | England | Longitudinal study, 12 years | 3937, 61.7 (7.9), male 1709 (43.4%) | “Whether you use the internet and/or email.” Yes or no. | cognitive function | Modified telephone interview for cognitive status (TICS), probable dementia or cognitive impairment (0–11) and non-impaired (12–27) | gender, centered date of birth, highest educational qualification achieved, 5-category social class, age of finishing formal education, income quintile, wealth quintile, ethnicity (white or non-white), and parental household structure, occupational classification and smoking. Retirement, participation in other activities as described above, activities of daily living, caring, homemaking, self-rated health, self-rated hearing, self-rated eyesight, marital status, diagnosed chronic diseases including psychiatric illness, depression (score of modified center for epidemiological studies scale), number of cigarettes smoked per day (0, 1–10, 11–19 or 20), vigorous, moderate and light exercise and cognitive function | Refer to no use, wave 2: RR (95%CI) 0.74 (0.61–0.89), wave 3: 0.78 (0.65–0.94), wave 4: 0.72 (0.60–0.86), wave 5: 0.64 (0.54–0.77), wave 6: 0.66 (0.56–0.79) |  |
| Almeida et al, 2012 [50] | Australia | Longitudinal cohort study, average follow up of 6.0 years (range: 6 months to 8.5 years) | 5506, 75.5 (4.2), all male | ‘How often do you use a personal computer?’ Possible answers were ‘never’, ‘every day’, ‘at least every week’, ‘less than every week’. We classified participants who answered ‘never’ as ‘computer no-users’ and those who offered any of the other three answers as ‘computer users’. | Cognitive function | Western Australian Data Linkage System (WADLS) International Classification of diseases tenth revision (ICD-10) | age, high school attainment, social network, and presence of depression and significant clinical morbidity | HR of dementia amongst computer users was HR = 0.62, 95%CI = 0.47–0.81 compared with non-users |  |
| d’Orsi et al, 2017 [51] | English | Cohort study, 10 years | 8238, 50 years or above, 3,713 (45.07) | responses to the statement “I use the internet or email”, recorded at 2002–03 and/or 2004–05 as a binary (yes/no) variable. | Cognitive function, | short-form IQCODE questionnaire, cut-off point of 3.5 | demographic (gender, age, marital status); and socioeconomic (education and wealth) variables, self-rated comorbidities (hypertension, diabetes, stroke, CHD and cancer), impaired mobility, depression and baseline cognitive function | Refer to no use, internet use HR 0.60301 (0.42–0.85) | Cox regression |
| Nakagomi et al, 2021 [52] | Japan | Cohort study, 3 years | 4232, 65 years or older, 1575 (37.2%) | Participants were asked: Have you used the Internet or e-mail in the past year? If yes, please indicate how often: a few times a month, a few times a week, almost every day. Internet use was categorized as “Not at all”, “Use a few times a month”, “Use a few times a week”, and “Use almost every day”. | Cognitive function | Dementia obtained via linkage to registries maintained by local municipal governments recorded under the Japanese LTCI system | sociodemographic factors (age, gender, education, household income (tertile), employment, marital status, living alone, population density), baseline ADL, and prior outcome values except for death, dementia, functional disability, and sedentary lifestyle | Refer to not use, use a few times a month OR 0.84 (0.53, 1.33), use a few times a week 0.69 (0.39, 1.21), use almost every day 0.85 (0.54, 1.32) |  |
| Quialheiro et al, 2021 [53] | Brazil | Cohort study, 10 years | 594, 60 years or above, 482 (38.6) | identified by the question, “Do you use the internet or e-mail?” and answered by the older adults with binary options (yes/no). | Cognitive function | MMSE, Older adults with no education and a score of 19 or less in the MMSE, or older adults with some education and a score of 23 or less in the MMSE were considered to have cognitive impairment | sex, age, years of education, household income, self-reported comorbidities (stroke, arthritis, cardiovascular disease, back pain, depression, diabetes, hypertension), and time. | Refer to no use, incidence rate ratio 0.30 (0.15–0.61). |  |
| Wen et al, 2023 [24] | China | Cross-sectional study | 13474, 61.50 (9.30), 6526 (48.43) | Internet use was considered the core explanatory variable, with 1 representing internet access and 0 otherwise. | Physical function | Activities of Daily Living (ADL) Scale.  No difficulty in any of the ADL items was scored as 0, and at least 1 difficulty was scored as 1 | Not adjust | Refer to no use, OR (95%CI): 0.48 (0.39-0.60) |  |
| Medeiros et al, 2012 [19] | Brazil | Cross-sectional study | 1656, aged 60 years or older, 598 (36.1) | ability to exchange online messages, able to send and receive online messages without difficulty; able to send and receive online messages with difficulty/unable | Physical function | ADL scale, no/mild functional dependence (inability/difficulty to perform 0-3 activities) and moderate/severe functional dependence (inability/difficulty to perform 4-15 activities) | demographic, socio-economic, health, behavioral and social factors | Those who were able to send and receive online messages without difficulty remained with a significantly lower prevalence ratio for moderate/severe functional dependence (prevalence ratios=0.61; 95%CI: (0.40-0.94). | Poisson regression |
| Liu et al, 2023 [55] | China | Cross-sectional study | 10325, 60.32(9.06), 5,698 (55.19%) | “Do you use mobile payments, such as Alipay and WeChat pay?” and “Do you use WeChat?”, using WeChat and mobile payments, only using WeChat, and neither. | Cognitive function | MMSE, cognitive impairment or no cognitive impairment | age, sex, education level, smoking status, drinking condition, living alone, physical activities, social activities, hypertension, diabetes, dyslipidemia, stroke, chronic lung diseases, emotional, nervous, or psychiatric problems, memory-related disease, hearing disorder | Refer to Use WeChat and mobile payments, Neither WeChat nor mobile payments OR 3.48 (2.27, 5.33) |  |
| Li et al, 2022 [54] | China | Cross-sectional study | 3020, 60+, 1378 (45.6%) | “Whether you use the internet or email, and how often do you use the internet”, yes or not. | Cognitive function | MMSE and MoCA with the diagnosis from experienced psychiatrists; MCI, dementia, and normal | age, gender, education, daily living information (hobby, physical activities, sleeping patterns, dietary preferences, smoking history, and consumption of tea and alcohol) as well as disease related information | Refer to not use, MCI: OR 0.478 (0.264–0.867), Dementia: OR 0.393 (0.093–1.665) |  |
| Liu et al, 2023 [31] | China | Cross-sectional study | Rural: 5868, 68.07 (6.1), 3083 (52.54%)  Urban: 2355, 68.33 (6.4), 1154 (49%) | Have you used the Internet in the last month? Those who answered no to the first question were assigned 1, indicating “never.” | Physical function and cognitive function | Basic ADL and brief Community Screening Instrument for Dementia | age, gender, marital status, literacy, employment status, wage, pension, subsidy, pension insurance, medical insurance, accessibility to physical examination services | Refer to no use, functional disability OR 1.491 (0.794-2.799), cognitive function OR 0.052 (0.011-0.260) |  |
